# Assessing the prevalence of spinal deformities and their clinical effects in adolescent Egyptian males: a cross-sectional study

**DOI:** 10.1186/s13018-025-06388-6

**Published:** 2025-11-19

**Authors:** Dalia G Mahran, Ahmed A. Khalifa, Esraa Tulib, Mahmoud Fouad Ibrahim, Osama Farouk

**Affiliations:** 1 Department of Public Health and Community Medicine, Faculty of Medicine, Assiut University, Assiut, Egypt; 2 Department of Family Medicine, Faculty of Medicine, Assiut University, Assiut, Egypt; 3 Orthopaedic Department, Assiut University Trauma Hospital, Assiut University, Assiut, Egypt; 4 Department of Family Medicine, Ministry of Health, Kharga, New Valley Egypt; 5 Orthopaedic and Traumatology Department, Qena Faculty of Medicine and University Hospital, South Valley University, Kilo 6 Qena-Safaga highway, Qena, 83523 Egypt

**Keywords:** Adolescents, Spinal deformities, School students, Prevalence, Kyphosis, Scoliosis

## Abstract

**Background:**

The primary objective of this study was to determine the prevalence of back deformities among Egyptian adolescent males. Secondary objectives included identifying associated factors and assessing any relationship with back symptoms.

**Methods:**

A cross-sectional study was conducted during the 2020–2021 academic year among male students aged 12–14 years attending preparatory and secondary schools in Al-Khargah city, Egypt. A total of 532 students, having a mean age of 12.9 ± 0.5 years, were enrolled from randomly selected government schools. Assessment included a structured self-administered questionnaire, clinical screening for spinal deformities, and radiographic confirmation for suspected cases.

**Results:**

The clinical prevalence of back deformities was 15.4%, with 13.5% having kyphosis and 1.9% scoliosis. Radiographic confirmation revealed structural deformities in 6.8% of participants, while 8.6% had postural kyphosis. Significant factors associated with clinical deformities included being underweight (OR 6.17), prolonged mobile phone use (> 4 h daily, OR 2.67), owning a mobile phone for > 3 years (OR 2.22), watching television regularly (OR 3.33), and reclining/sleeping during TV viewing (OR 2.08/1.98). Rare consumption of yogurt (OR 3.44) and cheese (OR 3.63) was also significantly associated with spinal deformities. Students with deformities reported substantially more back pain over the past three months (OR 2.69).

**Conclusion:**

Back deformities are relatively common among adolescent males in southern Egypt and are significantly associated with modifiable lifestyle and nutritional factors. Early screening and targeted school-based health education may help prevent progression and reduce associated morbidity.

**Supplementary Information:**

The online version contains supplementary material available at 10.1186/s13018-025-06388-6.

##  Introduction

Postural changes and spinal deformities in adolescents showed an increasing prevalence, leading some authors to call it a social epidemic, with a prevalence ranging between 30% and 80% as reported from evaluating various populations [1–3]. Such deformities might be attributed to congenital or acquired causes, including skeletal injuries, pathologies such as rickets or tuberculosis, and bad postures, for proper management, the primary cause should be investigated, as some of these are correctable [1, 3, 4].

The burden on the healthcare system for managing spinal deformities rises as the deformity and the related symptoms become more severe [5]. Furthermore, patients presenting with more advanced clinical symptoms are expected to have a higher incidence of disabilities and unemployment, with further economic burden [6, 7].

One of the preventable and modifiable risk factors for the development of spinal deformities in adolescents is severe improper posture, which, if not corrected, might lead to progression of adolescent spinal deformities with further compromise of function and quality of life, so detecting, understanding, and correcting postural deviations is crucial to preventing and managing such deformities [1, 8–10].

Screening programs, which have been recommended to avoid missing spinal deformities with a subsequent deformity progression, are a step toward early detection and management of spinal deformities in the younger population [11, 12]. The Scoliosis Research Society’s International task force reported that screening was effective in technical, clinical, program, and treatment efficacy [11]; furthermore, most scoliosis and child health societies support screening [12, 13]. Some studies reported that discontinuing the screening programs increased the rate of surgical intervention [14, 15].

Furthermore, it became apparent that identifying the prevalence and possible factors associated with spinal deformities in adolescents is a step toward developing targeted public health educational and preventive programs focusing on proper posture, adjusting personal habits, correcting modifiable risk factors, and spine care [16]. However, studies focusing on these issues are scarce in our area [8, 17, 18].

So, we carried out the current research with the primary objective of determining the prevalence of back deformities (scoliosis and kyphosis) among Egyptian adolescent males attending preparatory and secondary government schools. The secondary objectives were to identify the possible factors associated with such back deformities and to determine if these deformities were associated with back-related clinical symptoms.

## Materials and methods

### Study design and setting

The current cross-sectional study was conducted during the Egyptian preparatory and secondary government schools’ academic year 2020–2021 after obtaining approval from our local Ethical Committee (IRB No.17101276) and following the ethical considerations according to the Helsinki declarations. Furthermore, informed consent was obtained from all participants’ guardians. We followed STROBE guidelines for conducting and reporting the current study (Supplementary file 1) [19].

The study was conducted in one city (Al-Khargah), the capital city of the New Valley governorate in Upper (Southern) Egypt. At the time of the study, Al-Khargah had 21 preparatory schools (1251 students in the 2nd and 3rd grades) and nine secondary schools (819 students in the 1st grade). We randomly selected governmental schools (five preparatory and two secondary) after contacting the local authorities and explaining the study purpose; furthermore, classes were randomly selected using stratified random sampling to ensure representative inclusion of students after considering their school lesson schedule.

### Sample size and power calculation

The sample size calculation was performed using OpenEpi, Version 7, an open-source calculator for cross-sectional studies. Assuming a prevalence of spinal deformity of 21% based on a previously published study [8], with a 95% confidence level and 80% power, the required sample size was calculated separately for each grade. The total number of male students for the second and third preparatory grades was approximately 900. The calculated sample size was 340 students, which was increased by 20% (68 students) to account for potential dropouts, resulting in a final target of 408 students. For the first secondary grade, where the total number of male students was around 400, the required sample size was 156. This was also increased by 20% (30 students) for anticipated attrition, leading to a final sample of 186 students. Accordingly, the total estimated sample size needed for the study was 594 male students.

### Research participants, inclusion and exclusion criteria

We included male students from the second and third preparatory grades and first secondary grade at governmental schools (the usual age range for such grades is between 12 and 14 years old) who were willing to participate in the study (or approved by their parents or legal guardians). We excluded students who refused to participate, those with known syndromes affecting normal growth (e.g., dwarfism, Marfan syndrome, osteogenesis imperfecta), those who had previous spine issues (such as known traumatic, neurological, or infectious problems), and students who had other skeletal injuries or surgeries that might affect their posture (such as having a previous hip or lower limbs fractures leading to leg length discrepancy).

### Outcomes assessment (Questionnaire, clinical and radiological evaluation)

The outcomes assessment was divided into three sectors:

First is a self-administered questionnaire, which was designed for the current study after reviewing the relevant literature from other populations that investigated the same issue as we aimed to evaluate in the current study (some of the items were modified to accommodate our culture and financial constraints) [8, 20]. The questionnaire was divided into four sections (supplementary files 2 and 3):

1. Demographic data: age, residence, parents’ education.
2. Risk factors: bad postural habits, heavy school bags, mobile phone usage, TV watching habits, sports activities, sitting and writing positions, and nutritional factors.
3. Possible health impacts related to back issues: physical activity reduction, back pain (location and intensity), and school absenteeism.

Second, clinical evaluation:

1. General physical examinations: This was conducted in the school nurse’s room and included anthropometric measurements such as weight, height, and BMI, as well as determining if there were any obvious other limb deformities.
2. Clinical screening of spinal deformity (kyphosis or scoliosis). This was performed by one of the authors (E.T.), who was trained on how to assess the presence of spinal deformities by the following clinical assessment techniques:
  - Inspection (from the front, back and from the sides) and Adams forward-bending test (the child freely bends forward at the waist keeping the knees straight and arms together toward the floor, and the back parallel to the floor while the examiner looks along the axis of the spine) to observe any obvious deformities (uneven shoulders, rib hump, asymmetric back, body deviation) and inspection from the side after bending forward to detect if a kyphotic deformity is accentuated [12, 21, 22].
  - Visually assessing the range of motion (ROM) for cervical, thoracic, and lumbar spines was performed to detect any severe motion limitation. A crude clinical assessment of cervical, thoracic, and lumbar spine motion was performed. The cervical spine was assessed while the participant was standing. A good ROM was considered the ability to flex the neck so that the chin could touch the chest, extend the neck approximately 30 degrees beyond neutral, and perform lateral flexion to about 30 degrees to either side (ear approaches the shoulder). The lumbar spine was assessed while the participant stood, and the knees were extended. A good ROM was considered if the participant could bend forward so that the fingertips reach the ankles, extend the lumbar spine approximately 20° beyond neutral, and perform lateral flexion so that the fingertips reach up to the knees level. For the thoracic spine, rotation was assessed with the participant seated, arms crossed over the chest, and pelvis stabilized. A good ROM was defined as the ability to rotate the shoulders so that they point to approximately half the distance of the arc between the coronal and sagittal planes (about 45 degrees of rotation to either side) [12, 23–25].

Third, for students who showed deformities during the screening clinical examination, they were transferred to the orthopaedic department at the local university hospital to have radiographic evaluation to confirm spinal deformity was performed by obtaining a series of plain X-rays (posteroanterior and lateral views), which were evaluated by two of the authors (M.F.I. and O.F.), where the scoliosis was diagnosed if a Cobb angle of **≥** 10 degrees on standing posteroanterior radiographs was detected. On lateral radiographs, Kyphosis was defined as a thoracic kyphosis angle (Cobb angle T4-T12) of >40 degrees, and the diagnosis was confirmed based on the Sorensen criteria [21, 26, 27].

Based on the clinical and radiological evaluation, patients with clinically evident deformities were divided into either postural or structural spine deformities if the radiological evaluation proved negative or positive regarding the criteria mentioned above, respectively.

We conducted a pilot study on 30 participants (their data were not included in the final analysis) to evaluate the simplicity and the proper understanding of the study questionnaire, which improved in clarifying any unclear or ambiguous questions, and to calculate a rough estimate of the time required to collect the questionnaire.

Of the required sample size, 62 students (10.4%) refused to participate, leading eventually to the inclusion of 532 participants.

### Statistical analysis

Data analysis was performed using SPSS version 22. Descriptive statistics were reported as appropriate, mean ± standard deviation, median (range), and numbers (percentages). The Mann–Whitney U test was used to compare continuous variables between groups, while the Chi-square test was applied to assess associations between categorical variables. Logistic regression analysis was conducted to identify significant factors associated with back deformities, where variables were selected for inclusion based on clinical relevance and the significant associations in bivariate comparisons. In addition to univariate comparisons, a crude odds ratio (OR) with a 95% confidence interval (CI) was calculated to estimate the strength of association between back deformity and the presence of back pain. A p-value of < 0.05 was considered statistically significant.

## Results

### Demographic data

We evaluated 532 male students having a mean age of 12.9 ± 0.5 (ranging from 12 to 14), and 99.6% were urban area residents. The participants were distributed across the second and third preparatory and first secondary grades as 30.1%, 36.5%, and 33.4% respectively. The overall mean BMI was 20.47 ± 2.84 (ranging from 13.3 to 28.7), where the majority of students were within a normal weight range according to BMI percentiles, with 9 (1.7%) underweight, 410 (77.1%) normal weight, and 113 (21.2%) overweight. No students were classified as obese.

### Prevalence of back deformities

The prevalence of back deformity by clinical evaluation was 15.4% (82 participants), 72 (13.5%) having kyphosis, and 10 (1.9%) having scoliosis. However, the radiographic evaluation showed that only 36 (6.8%) had structural deformity, 26 (4.9%) had kyphosis (Scheuermann kyphosis), and 10 (1.9%) had scoliosis (adolescent idiopathic scoliosis), which indicated that 46 (8.6%) of the participants had postural kyphosis.

### Factors associated with back deformities

Clinical back deformity was significantly associated with lower BMI (19.57 ± 1.96 vs. 20.64 ± 2.94, *p* = 0.001), playing football (81.7% vs. 70.9%, *p* = 0.022), possessing Mobile Phone for more than three years and using it for more than four hours daily, (31.7% vs. 19.3%, *p* = 0.033) and (50.0% vs. 36.0%, *p* = 0.006), respectively. Furthermore, watching TV regularly and reclining/sleeping while watching were significantly associated with back deformity (89.0% vs. 70.9%, *p* = 0.001) and (15.9%/34.1% vs. 8.4%/19.1%, *p* = 0.026). Regarding dietary habits, rare yogurt or cheese consumption showed a significant association with back deformity (25.6% vs. 13.6%, *p* = 0.019) and (15.9% vs. 5.3%, *p* = 0.002), respectively (Table 1).

**Table 1:** Factors associated with the presence of back deformity

Variables			No deformity (*n* = 450, 100%)		Deformity (*n* = 82, 100%)		*P* value
			n	%	n	%	
Age		Median (range)	12.8 (12.0–13.9)		13.0 (12.1–14.0)		0.063^‡^
BMI		Median (range)	20.2 (13.3–28.7)		19.4 (15.7–23.9)		**0.001** ^‡^
Sports activities	Regularly playing sports	No	47	10.4	8	9.8	0.851^†^
		Yes	403	89.6	74	90.2	
	Type of sport	Football	319	70.9	67	81.7	**0.022** ^†^
		Others	84	18.7	7	8.5	
School bag carrying habits	How to carry the bag	On one shoulder	46	10.2	9	11.0	0.594^†^
		On both shoulders	396	88.0	63	76.8	
	Is the bag heavy?	Yes	248	55.1	43	52.4	0.566^†^
		No	194	43.1	29	35.4	
Body position habits	Writing position*	Poor	422	93.8	81	98.8	0.068^†^
		Good	28	6.2	1	1.2	
	Setting position*	Poor	391	86.9	70	85.4	0.709^†^
		Good	59	13.1	12	14.6	
	Parents give instructions to set straight	Yes	323	71.8	67	81.7	0.062^†^
		No	127	28.2	15	18.3	
	Studying in bed	Yes	92	20.4	14	17.1	**0.007** ^†^
		No	160	35.6	17	20.7	
		Sometimes	198	44.0	51	62.2	
Habits related to mobile phones	Owning a mobile phone	No	35	7.8	5	6.1	0.596^†^
		Yes	415	92.2	77	93.9	
	Duration of possessing a mobile phone	less than one year	178	39.6	24	29.3	**0.033** ^†^
		1–3 years	150	33.3	27	32.9	
		More than 3 years	87	19.3	26	31.7	
	Daily mobile phone use	Less than 2 h daily	158	35.1	15	18.3	**0.006** ^†^
		2–4 h daily	95	21.1	21	25.6	
		> 4 h daily	162	36.0	41	50.0	
	Parents’ control over using mobile phones	Yes	155	34.4	23	28.0	0.141^†^
		No	37	8.2	12	14.6	
		Sometimes	223	49.6	42	51.2	
Habits related to watching TV	Watching TV regularly	No	131	29.1	9	11.0	**0.001** ^†^
		Yes	319	70.9	73	89.0	
	Duration of watching TV	Less than 2 h daily	274	60.9	62	75.6	0.224^†^
		2–4 h daily	20	4.4	8	9.8	
		> 4 h daily	25	5.6	3	3.7	
	Position while watching TV	Sitting	195	43.3	32	39.0	**0.026** ^†^
		Reclining	38	8.4	13	15.9	
		Sleep	86	19.1	28	34.1	
	Parent’ control on watching TV	Yes	98	21.8	13	15.9	**0.006** ^†^
		No	116	25.8	41	50.0	
		Sometimes	105	23.3	19	23.2	
Dietary habits	Drink milk	Daily	80	17.8	14	17.1	0.325^†^
		Twice a week	47	10.4	4	4.9	
		At distant intervals	186	41.3	33	40.2	
		Very rarely	137	30.4	31	37.8	
	Eat yogurt	Daily	90	20.0	9	11.0	**0.019** ^†^
		Twice a week	50	11.1	7	8.5	
		At distant intervals	249	55.3	45	54.9	
		Very rarely	61	13.6	21	25.6	
	Eat cheese	Daily	369	82.0	55	67.1	**0.002** ^†^
		Twice a week	39	8.7	8	9.8	
		At distant intervals	24	5.3	13	15.9	
		Very rarely	18	4.0	6	7.3	

^†^The chi-square (χ2) test was used for comparison. ^‡^Mann Whitney U test were used for comparison. Significance defined by *p* < 0.05 (indicated by bold numbers)

N, number; BMI, body mass index; SD, standard deviation; n, number

After performing a multivariate regression analysis based on the significantly associated variables, we found that the followings variables are independently associated with back deformity (Table 2), being underweight (OR  6.171, 95% CI 1.011–37.682) or normal weight (OR  4.836, 95% CI 1.906–12.267), possessing a mobile phone for more than three years (OR  2.216, 95% CI 1.203–4.084) and using it more than four hours daily (OR  2.666, 95% CI 1.419–5.009). Watching TV regularly (OR  3.331, 95% CI 1.619–6.855) and reclining/sleeping positions (OR  2.085/1.984). Rare yogurt or cheese consumption (OR  3.443, 95% CI 1.478–8.021) and (OR  3.634, 95% CI 1.748–7.556), respectively.

**Table 2:** Multivariate logistic regression analysis for variables associated with the development of back deformity among the studied participants

Variables		*n*	Multivariate analysis		
			*P* value	OR	95% CI
BMI	Under weight	9	**0.049**	6.171	1.011–37.682
	Normal	410	**0.001**	4.836	1.906–12.267
	Over weight	113	ref		
Duration of having a mobile phone	less than one years	202	ref		
	1–3 years	177	0.338	1.335	0.739–2.411
	More than 3 years	113	**0.011**	2.216	1.203–4.084
Daily mobile phone use	Less than 2 h daily	173	ref		
	2–4 h daily	116	**0.020**	2.328	1.145–4.735
	> 4 h daily	203	**0.002**	2.666	1.419–5.009
Watching TV regularly	No	140	ref		
	Yes	392	**0.001**	3.331	1.619–6.855
Position while watching TV	Sitting	227	ref		
	Reclining	51	**0.049**	2.085	1.002–4.336
	Sleep	114	**0.018**	1.984	1.125–3.498
Parents’ control to watching TV	Yes	111	0.422	0.733	0.344–1.563
	No	157	**0.030**	1.953	1.067–3.576
	Sometimes	124	ref		
Type of sport	Football	386	**0.026**	2.520	1.116–5.693
	Others	91	ref		
Studying in bed	Yes	106	0.108	0.591	0.311–1.122
	No	177	**0.003**	0.413	0.229–0.742
	Sometimes	249	ref		
Eat yogurt	Daily	99	ref		
	Twice a week	57	0.529	1.400	0.492–3.987
	At distant intervals	294	0.125	1.807	0.849–3.846
	Very rarely	82	**0.004**	3.443	1.478–8.021
Eat cheese	Daily	424	ref		
	Twice a week	47	0.441	1.376	0.611–3.099
	At distant intervals	37	**0.001**	3.634	1.748–7.556
	Very rarely	24	0.103	2.236	0.851–5.878

N, number; CI, Confidence interval; OR, Odds ratio; Significance defined by *p* < 0.05 (indicated by bold numbers)

### Association between back deformities and clinical symptoms in the past three months

Although there was no difference between the two groups regarding the prevalence of headache or neck pain, p-values were 0.066 and 0.561, respectively. However, participants who had back deformity showed higher prevalence of back pain (sometimes: 59.8% vs. 41.6%; and a lot: 18.3% vs. 15.3%, *p* < 0.001), but there was no difference regarding back pain resulting in more often school absence (9.8% vs. 10.9% ) or hindered daily activities (4.9% vs. 11.1%), *p* = 0.214 (Table 3). There was a significant association between back deformity and the frequency of back pain (*p* < 0.001). Participants with back deformity had approximately 2.7 times higher odds of reporting back pain (“a lot” or “sometimes”) compared to those without deformity (OR  2.69; 95% CI 1.55–4.69).

**Table 3:** The possible health effects of back deformity among the participants in the past three months

Clinical symptoms		No deformity (*n* = 450, 100%)		Deformity (*n* = 82, 100%)		*P* value
		*n*	%	*n*	%	
Headache	Yes, a lot	109	24.2	20	24.4	0.066
	Sometimes	185	41.1	44	53.7	
	Rarely	86	19.1	7	8.5	
	No	70	15.6	11	13.4	
Neck pain attacks	Yes, a lot	74	16.4	9	11.0	0.561
	Sometimes	209	46.4	42	51.2	
	Rarely	50	11.1	11	13.4	
	No	117	26.0	20	24.4	
Neck pain intensity	I can handle it	272	60.4	49	59.8	0.738
	It causes absence from school	42	9.3	10	12.2	
	It hinders daily activities (walking, praying, studying)	19	4.2	3	3.7	
Back pain attacks	Yes, a lot	69	15.3	15	18.3	**< 0.001**
	Sometimes	187	41.6	49	59.8	
	Rarely	194	43.1	18	22.0	
Back pain intensity	I can handle it	351	78.0	69	84.1	0.214
	It causes absence from school	49	10.9	8	9.8	
	It hinders daily activities (walking, praying, studying)	50	11.1	4	4.9	

The chi-square (χ^2^) test was used for comparison. Significance defined by *p* < 0.05 (indicated by bold numbers)

## Discussion

The fundamental findings of the current study included an overall clinically evident spinal deformity prevalence of 15.4%, where kyphosis was higher than scoliosis, 13.5% vs. 1.9%, respectively. Furthermore, all the scoliosis cases were structural, as proved by radiological examination, while structural kyphosis was detected in only 4.9% of cases, and 8.6% had postural kyphosis. Besides being under or of normal weight, bad habits related to using mobile phones and watching TV were associated with increased prevalence of spinal deformity. Moreover, experiencing attacks of back pain was significantly associated with the presence of spinal deformity.

Regarding the prevalence of spinal deformities in the current study in comparison to previous studies, a similar scoliosis prevalence of 1.5% was reported by Penha et al., who conducted the study on 2562 Brazilian adolescents aged between 10 and 14 years [28]. In a study by Elshazly et al. on 1117 Saudi students (who have nearly similar characteristics to the current study population) having a mean age of 12.05 ± 2.2, the clinically proven scoliosis prevalence was as low as 0.81%, which is lower than the current study [18]. On the contrary, a study by Scaturro et al. of 428 Italian students (aged between 11 and 14 years old) reported a clinically evident scoliosis prevalence of 15.4%, nearly eight times the prevalence we found in the current study [29]. A more recent study conducted on 591 Saudi students by Al-Assiri et al. reported a scoliosis suspicious based on scoliometer measurements of 29.4%, however, this possibly higher prevalence could be attributed to two factors, the higher age range (12 to 18 years old), more than 50% of the participants were females, and there was no radiological confirmation of the suspected deformities [17].

Concerning the kyphosis deformity prevalence, we reported a clinically evident kyphosis prevalence of 13.5%; of those, 4.9% had structural kyphosis, while 8.6% had a postural kyphosis. In a study by Akhter et al., who evaluated 150 Pakistani students with a mean age of 11.3 years (range between 7 and 14), only 40% were males. Kyphosis was assessed clinically using a flexi curve ruler; they reported a kyphosis prevalence of 38.7% [30], which is considerably higher than the current study. In contrast, a systematic review and meta-analysis by Taleschian-Tabrizi et al., which included 84,195 students (consisting of 46.6% boys and 53.4% girls) having a mean age of 12.71 ± 1.18 years as reported from 18 studies, the kyphosis and scoliosis prevalence was 13.1% and 2.6% respectively, which coincides with our results, they also reported that the deformity prevalence was higher in girls [31].

A crucial point worth mentioning is that, related to the screening performed first by clinical examination, followed by radiographic evaluation for positive cases. This protocol was followed in the current study, which was also reported in previous studies [29]. Per the Guideline for adolescent scoliosis screening in China, they recommended initial clinical screening which might be performed at home or school using various clinical methods (visual inspection, forward bending test, and Portable electronic spinal scoliosis screening tool), and if positive, the student should be refereed for radiological evaluation, based on which the management needed will be prescribed [12].

Various factors associated with spinal deformity development in adolescents were reported in many previous studies, indicating that it is a multifactorial issue, and searching for all possible causes is paramount for designing a proper prevention program.

We found that under- or normal-weight students had a higher chance of developing spinal deformities, OR was 6.171 and 4.836, respectively. However, the results in the literature were controversial; some studies aligned with our findings [32–34], while others reported the opposite [29, 35, 36]. Some authors noted that the relationship between higher BMI and spinal deformities could be attributed to the pressure exerted by weight on the anterior vertebral growth plates, preventing them from properly growing, leading eventually to spinal deformity, including kyphosis [37, 38].

Students’ daily habits were significantly associated with developing spinal deformities, such as longer periods of using mobile phones or watching TV, especially while posing incorrect postures [39, 40].

Although Din et al., who studied 374 Malaysian female students having a mean age of 10.8 ± 0.7 years and a scoliosis prevalence of 5.6% reported no association between scoliosis and the time spent in front of screens or while using smartphones (*p* = 0.219) [20], the opposite was reported in the current study where a significant association between spinal deformities and prolonged usage of smartphone (OR 2.666, *p* = 0.006), watching TV regularly (OR 3.331, *p* = 0.001), and reclining/sleeping positions (OR 2.085, *p* = 0.026). Moreover, a matched case-control study by Khadour et al. included 1102 Syrian adolescents with a mean age of 13.5 ± 1.08 years, and 56.3% were girls. The authors reported a significant association between bad posture and personal habits (inappropriate sitting position) and scoliosis development [41]. In another study from China by Zheng et al., which investigated 11,024 primary school students, concluded that spending more time using computers was a predictor of scoliosis development [42].

Conversely, in line with the current study, Scaturro et al. observed that playing sports, especially high-risk sports, for more than three hours was associated with an increased risk of developing scoliosis [29].

Dietary habits and deficiencies were a matter of concern related to their association with spinal deformities. Dop et al. investigated 134 children aged between 5 and 18 years to identify the factors associated with spinal deformities; they reported a prevalence of scoliosis and kyphosis of 21% and 7.5% respectively. Among several investigated variables, the authors identified vitamin D, calcium, magnesium deficiencies, and inadequate diets as possible risk factors associated with the development and progression of spinal deformities [1]. Although we did not carry out a detailed nutritional status evaluation, we found that students who rarely consumed yogurt or cheese were about three times more likely to develop spinal deformities (ORs were 3.443 and 3.634, respectively). However, as reported with other variables, this finding was a matter of debate among studies, where some authors reported a positive correlation between spinal deformities and dietary habits or nutritional status, such as a study by Godzialska et al., who reported that vitamin D deficiency could be involved in the pathogenesis of AIS [43]. On the contrary, Asakura et al. and Din et al. reported no relationship between dietary habits, including vitamin D intake, and spinal deformity development [20, 44].

A crucial issue related to the spinal deformities in adolescents is the development of clinical symptoms secondary to the deformities, such as back pain. A systematic review by Théroux et al. included 598 adolescents with scoliosis; the prevalence of low back pain ranged from 34.7 to 42.0% [45]. Furthermore, although Scaturro et al. reported no differences regarding the incidence of back pain between adolescents who had spinal deformities compared to those who did not, those with spinal deformities had significantly longer duration (more than three hours) of back pain daily, *p* < 0.001 [29]. In the current study, we found a higher prevalence of back pain among students with spinal deformities (*p* < 0.001); they were approximately 2.7 times more likely to experience back pain if there was a spinal deformity.

Although one major strength of the current study is that it is among the few studies to evaluate the prevalence of spinal deformities in adolescent males in Egypt, we admit several limitations that might originate from its cross-sectional nature, being performed on a specific population, and its concise primary objective.

First, some evaluation aspects were deficient, such as a lack of precision when reporting the ROM, and we did not provide the exact angles measured during the radiographic assessment. However, the precision of visual evaluation of the deformity was reported to reach up to 85%, and when coupled with the radiographic assessment, the accuracy is even higher [12, 22]. Furthermore, a detailed investigation of the nutritional factors, such as Vitamin D levels, was not possible due to logistical and financial constraints.

Second, the current study lacks gender (no females were evaluated) and ethnic (only Egyptians) diversity. The reason behind excluding females is that nearly all female students refused to participate during the pilot study, and we admit that this gender bias in students’ inclusion might affect the results’ generalizability. However, various variables were discussed in the literature on a larger and diverse population, such as the studies derived from the evaluation of the Multi-Ethnic Alignment Normative Study (MEANS) database, which included a population from a Middle Eastern and North African population (Tunesia) whose characteristics might be similar to the population included in the current study [46–49]. Sardar et al. reported that ethnicity affected the incidence of lumbar lordosis and thoracic kyphosis, where the normal Arabo-Bèrbère (Middle Eastern and North African) and Caucasian populations reported higher values compared to their Asian counterparts. However, all had similar pelvic incidence, pelvic tilt, and sacral slope. The authors noted that lumbar lordosis correlates with thoracic kyphosis; they concluded that thoracic kyphosis varies based on race [47].

Third, there is no investigation of other parameters that might affect the incidence of low back pain and could be closely related to the spinal deformities. In another study on the same MEANS database by Hasegawa et al. investigated factors affecting the pelvic incidence, which is closely related to spinal sagittal deformities (might also lead to the development of sacroiliac joint and low back pain), and they reported that it is affected by gender, ethnicity, and age [46].

Fourth, further studies on the MEANS database found that the participant showed some compensatory mechanisms to correct the sagittal imbalance, such as pelvic retroversion, increased cervical lordosis, and knee flexion [48, 49]. We believe that such compensatory mechanisms should have been evaluated in the current study to delineate any spinal sagittal deformities that might be concealed by such a compensatory mechanism.

Fifth, the back pain was not evaluated in a systematic way or by using specific instruments as used in previous studies (such as the Oswestry low back pain disability questionnaire, the Japanese Orthopaedic Association Back Pain Evaluation Questionnaire, the Scoliosis Research Society (SRS)-22r questionnaire, and the visual analog scale (VAS)) [50]. Furthermore, the association between back pain and the presence of back deformity was evaluated using a crude (unadjusted) odds ratio estimation, which does not account for potential confounders; however, as this was not the primary objective of the study, we did not perform multivariate logistic regression to investigate other variables that might be associated with back pain.

Sixth, we did not perform univariate regression screening before multivariate analysis, which could have helped refine variable selection. However, we overcame this by including only factors that showed significant or near-significant associations in preliminary bivariate tests. Lastly, the higher rate of dropouts compromised the study’s power.

## Conclusion

The overall prevalence of back deformity among Egyptian adolescent male students was comparable to the literature, with kyphosis deformity being presented more than scoliosis. Furthermore, about half of the kyphosis cases were proven to be postural after radiological evaluation. Various risk factors were identified as possibly associated with spinal deformities, such as low BMI, improper habits related to using mobile phones and watching TV, and inadequate diet. The presence of spinal deformities was significantly associated with back pain attacks.

The results obtained from the current study could serve as a step toward a nationwide screening program by utilizing simple clinical and radiological evaluation. Furthermore, preventive measures could be initiated by dealing with the modifiable risk factors.

## Supplementary Information

Below is the link to the electronic supplementary material.

Supplementary Material 1

Supplementary Material 2

Supplementary Material 3

## Data Availability

No datasets were generated or analysed during the current study.
