# Supplementary material for: Assessing the prevalence of spinal deformities and their clinical effects in adolescent Egyptian males: a cross-sectional study
Source: J Orthop Surg Res. 2025 Nov 19;20:1011. doi: 10.1186/s13018-025-06388-6 (PMC12628987; doi:10.1186/s13018-025-06388-6)
Supplement: Supplementary file 2 — Supplementary Material 2 [file 13018_2025_6388_MOESM2_ESM.pdf]

Department of Family Medicine

Faculty of Medicine

Questionnaire of Spinal Deformities, Determinants and Their Impact on Lifestyle among Adolescent

1. This special questionnaire aims to find out the prevalence of spinal deformities, their determinants and their impact on the lifestyle among adolescent students in the city of Kharga.
2. Your participation in this research is voluntary, knowing that this data will be treated in strict confidentiality and will only be used in the field of scientific research.

Please answer the following questions. Please complete all questions

	(optional) Name	- 1
	Age	- 2
1- Rural 2- Urban	Residence place	- 3
Illiterate / Literacy certificate / Primary / Preparatory / Secondary / Bachelor's / Master's or Doctorate	Father's highest educational level	- 4
Farmer / Unskilled worker / Craftsman / Professional (doctor, engineer, teacher, etc.) / Employee / Retired / Unemployed / Other	Father's occupation	- 5
Illiterate / Literacy certificate / Primary / Preparatory / Secondary / Bachelor's / Master's or Doctorate	Mother's highest educational level	- 6
Unskilled worker / Craft work (e.g., sewing) / Professional / Employee / Retired / Housewife / Other	Mother's occupation	- 7

• Yes / No	Do you own a smartphone	- 8
If yes, please answer questions A1 to A4		
<1 year -1<br>1 to 3 years -2	duration of ownership	A1

> 3 years -3		
< 2 hours -1<br>2 to 4 hours -2<br>> 4 hours -3	Daily smartphone use	A2
Making calls -1<br>Texting and communication with friends -2<br>Browsing social media -3<br>For studying -4<br>news -5<br>Games and entertainment -6<br>For passing time only -7<br>others -8	Main purpose of use<br>you can select more than one)<br>(answer	A3
yes -1<br>no -2<br>sometimes -3	Do your parents limit your smartphone use?	A4
yes -1<br>no -2	Do you watch TV at home?	- 9
If yes, please answer questions from B1 to B3		
<2 hours -1<br>2 to 4 hours -2<br>> 4 hours -3	Daily TV watching time	B1-
Sitting -1<br>Reclining -2<br>Lying down -3	Posture during TV watching	B2
yes -1<br>no -2<br>sometimes -3	Do your parents limit your TV watching?	B3
yes -1<br>no -2	Do you have a computer or laptop?	10
If yes, answer C1		
<2 hours -1<br>2 to 4 hours -2<br>> 4 hours -3	Daily computer/laptop use	C1
yes -1<br>no -2	Do you practice sports?	11
If yes, please answer D1 and D2		
....	Type of sport	D1
yes -1<br>no -2	Do your parents encourage sports?	D2
Walking -1<br>Bicycle -2<br>Bus or car -3<br>Others -4	How do you go to school?	12
yes -1<br>no -2	Do you carry a school bag?	13

One shoulder -1<br>Both shoulders -2	How do you carry your school bag?	14
yes -1<br>no -2	Do you consider your school bag as heavy?	15
....................................(specify) yes -1<br>no -2	Do you work during summer vacation?	16
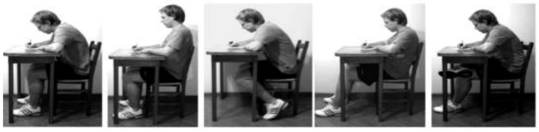	Posture during writing	17
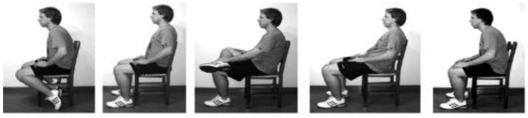	Posture during sitting	18
Yes - 1<br>no - 2	Do your parents advise you to sit upright?	19
yes -1<br>no -2<br>sometimes -3	Do you study on bed?	20
< 6 hours -1<br>6 to 8 hours -2<br>> 8 hours -3	Daily sleeping hours	21
daily -1<br>twice weekly -2<br>Occasionally -3<br>Rarely -4	Do you drink milk?	22
daily -1<br>twice weekly -2<br>Occasionaly -3<br>Rarely -4	Do you eat yogurt?	23
Daily - 1<br>Twice weekly - 2<br>Occasionaly - 3<br>Rarely - 4	Do you eat cheese?	24
Yes - 1<br>No - 2	Do you usually get sun exposure?	25
Frequently -1<br>sometimes -2<br>rarely -3<br>no -4	Did you experience headaches in last 3 months?	26
Frequently -1<br>sometimes -2<br>rarely -3<br>no -4	Neck pain in last 3 months?	27
tolerable -1<br>Causes absence from school -2<br>Limits daily activities -3	If yes, describe neck pain severity	28

Frequently -1<br>sometimes -2<br>rarely -3<br>no -4	Back pain in last 3 months?	29
tolerable -1<br>Causes absence from school -2<br>Limits daily activities -3	If yes, describe back pain severity	30

Examination sheet

1- General examination: 1- Normal 2- abnormality:…………………….

2- Skeletal deformities of body joints and syndromes affecting normal growth as dwarfism:

-No

- Yes:

....

....

Back Examination:

The examination will be included in a table and an illustration of back examination will be included in the sheet

Pain: 1-present 2-Absent

Inspection: Inspect patient from all angles

- Front: Posture of head and neck – symmetry / abnormal position

-Symmetry of shoulders – note any malalignment: 1-present 2-Absent

- Side

-Cervical lordosis – assess for hyperlordosis: 1-present 2-Absent

-Thoracic kyphosis: 1-present 2-Absent

-Lumbar lordosis – assess for hyperlordosis: 1-present 2-Absent

- Behind

-Scars: 1-present 2-Absent

-Wasting: 1-present (………………) 2-Absent

-Scoliosis: 1-present 2-Absent

-Abnormal hair growth – spina bifida: 1-present 2-Absent

Skin pigmentation (Café Au Lait patches)

Move

1-Thoracic spine

- -Thoracic rotation– sit the patient down, with arms crossed across chest and ask to turn side to side

Abnormality or limitation:

1-present 2-Absent

2-Lumbar spine

*Assess active movements:

- Flexion: 1- Normal 2- limitation
- Extension: 1- Normal 2- limitation
- Lateral flexion: 1- Normal 2- limitation
- *Special tests*

Adams forward-bending test:

 This data will be treated with strict confidentiality and will only be used in the field of scientific research.
