# Supplementary material for: Assessing the prevalence of spinal deformities and their clinical effects in adolescent Egyptian males: a cross-sectional study
Source: J Orthop Surg Res. 2025 Nov 19;20:1011. doi: 10.1186/s13018-025-06388-6 (PMC12628987; doi:10.1186/s13018-025-06388-6)
Supplement: Supplementary file 3 — Supplementary Material 3 [file 13018_2025_6388_MOESM3_ESM.pdf]

قسم طب الاسرة

كلية الطب

- رقم الاستمارة: | | | | |

استبيان تشوهات العمود الفقري ومحدداتها وتاثيرها علي نمط الحياة بين الطلبة المراهقين

- هذا الاستبيان خاص يهدف الى معرفة مدى انتشار تشوهات العمود الفقري ومحدداتها وتأثيرها على نمط الحياة بين الطلبة المراهقين بمدينة الخارجة
- مشاركتك في هذا البحث تطوعية علما بأن هذه البيانات سوف تعامل بسرية تامة ولن تستخدم الا في مجال البحث العلمي

من فضلك أجب على الأسئلة التالية مع رجاء استكمال جميع الأسئلة

1 -	الاسم (اختياري)	
2 -	العمر	
3 -	محل الاقامة	1-ريف 2- حضر:
4 -	ما هي أعلى مرحلة تعليمية اتمها والدك (اعلى شهادة حصل عليها)؟	1- غير متعلم<br>2- محو أمية<br>3- ابتدائية<br>4- اعدادية<br>5- الثانوية العامة/الفنية/ الأزهرية<br>6- بكالوريوس/ ليسانس<br>7- ماجيستير/ دكتوراة
5 -	ماذا يعمل والدك؟؟	1- مزارع<br>2- عامل غير ماهر (عامل / بائع / حارس ....الخ)<br>3- عمل حرفي (ميكانيكي / نجار.... الخ)<br>4- عمل مهني (طبيب ، مهندس ، صيدلي ، مدرس ، محامي ...)<br>5- موظف 6- على المعاش<br>7-لا يعمل 8- أخرى (تذكر..........................)
6 -	ما هي أعلى مرحلة تعليمية اتمتها والدتك (اعلى شهادة حصلت عليها)؟	1-غير متعلم<br>2- محو أمية<br>3-ابتدائية<br>4-اعدادية<br>5- الثانوية العامة/الفنية/ الأزهرية<br>6- بكالوريوس/ ليسانس<br>7- ماجيستير/ دكتوراة
7 -	ماذا تعمل والدتك؟؟	1- عاملة غير ماهرة (عاملة في مدرسة / بائعة ......الخ)<br>2- عمل حرفي (مثل الحياكة)<br>3- عمل مهني (طبيبة ، مهندسة ، صيدلانية ، مدرسة.... الخ)<br>4- موظفة 5- على المعاش<br>6- ربة منزل 7- أخرى (تذكر...........................)

8 -	هل تمتلك هاتف ذكي:	1- نعم ٢- لا
في حالة الإجابة بنعم أجب الأسئلة من A4 -A1		
A1	منذ متى تمتلك هاتف ذكي؟	2- اقل من سنة<br>3- من سنة-3سنوات<br>4- أكثر من 3 سنوات
A2	ما معدل استخدامك اليومي للهاتف الذكي ؟	1- أقل من ساعتين يوميا<br>2- من ساعتين- 4 ساعات<br>3- أكثر من 4 ساعات

A3	ما الهدف الرئيسي لاستخدامك الهاتف الذكي ؟<br>(يمكن اختيار اكثر من اجابة)	1- اجراء المكالمات الضرورية<br>2- التواصل مع الاصدقاء وارسال الرسال النصية<br>3- تصفح مواقع التواصل الاجتماعي مثل الفيسبوك وتويتر<br>4- الحصول على معلومات للدراسة<br>5- مطالعة الأخبار/ الاحوال الجوية/ ..الخ<br>6- العاب ترفيهية<br>7- فقط لتمضية الوقت بدون هدف معين<br>8- أخرى (تذكر ....)
A4	هل يمنعك والديك من استخدام الهاتف لفترات طويلة؟	1- نعم<br>2- لا<br>3- احيانا
9 -	هل تشاهد التلفاز في المنزل؟	1- نعم<br>2- لا
	في حالة الإجابة بنعم أجب الأسئلة من B1 – B3	
B1-	كم من الوقت تقضيه وانت تشاهد التلفاز؟	1- اقل من ساعتين يوميا<br>2- من ساعتين – 4 ساعات<br>3- اكثر من 4 ساعات
B2	كيف يكون وضعك اثناء مشاهدة التلفاز؟	1- جالسا<br>2- متكئا<br>3- نائما
B3	هل يمنعك والديك من مشاهدة التلفاز ؟	1- نعم<br>2- لا<br>3- احيانا
10	هل لديك كمبيوتر او لاب توب في المنزل؟	1- نعم<br>2- لا
	3- في حالة الإجابة بنعم أجب C1	
C1	ما معدل استخدامك للكمبيوتر او اللاب توب؟	1- اقل من ساعتين يوميا<br>2- من ساعتين -4 ساعات<br>3- اكثر من 4 ساعات
11	هل تمارس الرياضة؟	1- نعم<br>2- لا
	في حالة الإجابة بنعم أجب D1&D2	
D1	ما نوع الرياضة التي تمارسها؟	....
D2	هل يشجعك والديك على ممارسة الرياضة؟	1- نعم<br>2- لا
12	كيف تذهب الي المدرسة؟	1- سيرا على الاقدام<br>2- بالدراجة<br>3- بالاتوبيس او سيارة الاسرة أو أخرى
13	هل تحمل الحقيبة المدرسية؟	1- نعم<br>2- لا
14	كيف تحمل الحقيبة المدرسية؟	1- علي كتف واحد<br>2- على الكتفين
15	هل حقيبتك المدرسية ثقيلة الوزن؟	1- نعم<br>2- لا
16	هل تعمل في الاجازة الصيفية؟	1- نعم (اذكر نوع العمل)....................................<br>2- لا
17	كيف يكون وضعك اثناء الكتابة؟	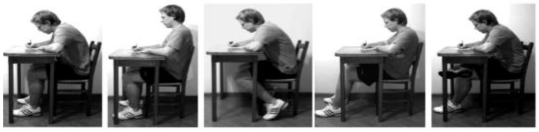
18	كيف يكون وضعك اثناء الجلوس؟	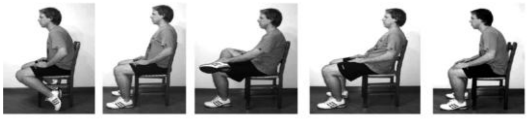
19	هل ينصحك والداك بضرورة الجلوس باستقامة، وإبقاء الظهر مستقيما؟	1 - نعم ٢- لا
20	هل تقرأ دروسك على الفراش؟	1- نعم<br>2- لا<br>3- احيانا
21	كم عدد ساعات نومك يوميا؟	1- اقل من 6 ساعات

		2- من 6-8 ساعات<br>3- اكثر من 8 ساعات
22	هل تشرب الحليب ؟	1- يوميا<br>2- مرتين اسبوعبا<br>3- علي فترات متباعدة<br>4- نادرا
23	هل تتناول زبادي ؟	1- يوميا<br>2- مرتين اسبوعبا<br>3- علي فترات متباعدة<br>4- نادرا
24	هل تأكل الجبنة ؟	5- يوميا<br>6- مرتين اسبوعبا<br>7- علي فترات متباعدة<br>8- نادرا
25	هل تتعرض للشمس في العادة؟	1 - نعم ٢- لا
26	هل عانيت من الصداع في الاشهر الثلاثة الماضية؟	1- نعم كثيرا<br>2- احيانا<br>3- نادرا<br>4- لا
27	هل تعاني من الام في الرقبة في الاشهر الثلاثة الماضية؟	1- نعم كثيرا<br>2- احيانا<br>3- نادرا<br>4- لا
28	في حالة وجود الم بالرقبة<br>ما مدى شدة الالم؟	1- استطيع تحمله<br>2- يتسبب في الغياب من المدرسة<br>3- يعيق النشاط اليومي (المشي- الصلاة- المذاكرة)
29	هل تعاني من الام في الظهر في الاشهر الثلاثة الماضية؟	1- نعم كثيرا<br>2- احيانا<br>3- نادرا<br>4- لا
30	في حالة وجود الم بالرقبة او الظهر<br>ما مدى شدة الالم؟	1- استطيع تحمله<br>2- يتسبب في الغياب من المدرسة<br>3- يعيق النشاط اليومي (المشي- الصلاة- المذاكرة)

Examination sheet

...............:Hieght

...............:Weight

1- General examination: 1- Normal 2- abnormality:…………………….

2- Skeletal deformities of body joints and syndromes affecting normal growth as dwarfism:

-No

- Yes:

....

....

Back Examination:

Pain: **1-present** **2-Absent**

Inspection: **Inspect the patient from all angles**

- Front: Posture of head and neck – symmetry / abnormal position

-Symmetry of shoulders – note any malalignment: 1-present 2-Absent

- Side

-Cervical lordosis – assess for hyperlordosis: 1-present 2-Absent

-Thoracic kyphosis: 1-present 2-Absent

-Lumbar lordosis – assess for hyperlordosis: 1-present 2-Absent

- Behind

-Scars: 1-present 2-Absent

-Wasting: 1-present (………………) 2-Absent

-Scoliosis: 1-present 2-Absent

-Abnormal hair growth – spina bifida: 1-present 2-Absent

Skin pigmentation (Café Au Lait patches)

Move

1-Thoracic spine

- -Thoracic rotation– sit the patient down, with arms crossed across chest and ask to turn side to side

Abnormality or limitation:

1-present 2-Absent

2-Lumbar spine

*Assess active movements:

- Flexion: 1- Normal 2- limitation
- Extension: 1- Normal 2- limitation
- Lateral flexion: 1- Normal 2- limitation
- *Special tests*

Adams forward-bending test:
